# Intraperitoneal Treatment of Cambinol, a Synthetic SIRT1 and SIRT2 Inhibitory Compound, Exacerbates *Brucella abortus* 544 Burden in the Spleens of Institute of Cancer Research Mice

**DOI:** 10.3390/microorganisms12122533

**Published:** 2024-12-09

**Authors:** Alisha Wehdnesday Bernardo Reyes, Tran Xuan Ngoc Huy, Trang Thi Nguyen, Said Abdi Salad, Ched Nicole Turbela Aguilar, Wongi Min, Hu Jang Lee, Suk Kim

**Affiliations:** 1 Department of Veterinary Paraclinical Sciences, College of Veterinary Medicine, University of the Philippines Los Baños, College, Laguna 4031, Philippines; abreyes4@up.edu.ph; 2 Microbial Research Division, UPLB Zoonoses Center, University of the Philippines Los Baños, College, Laguna 4031, Philippines; 3 Institute of Animal Medicine, College of Veterinary Medicine, Gyeongsang National University, Jinju 52828, Republic of Korea; txn.huy@hutech.edu.vn (T.X.N.H.); nguyentrang29071996@gmail.com (T.T.N.); dheerow2323@gmail.com (S.A.S.); cntaguilar24@gmail.com (C.N.T.A.); wongimin@gnu.ac.kr (W.M.); hujang@gnu.ac.kr (H.J.L.)

**Keywords:** *B. abortus*, cambinol, interleukin 10, splenic bacterial burden, sirtuins

## Abstract

Our preliminary data using bone marrow-derived macrophages (BMDMs) collected from ICR mice treated with anti-sirtuin (anti-SIRT) 1 antibody showed that *Brucella* uptake was significantly attenuated. We then further investigated the effect of an inhibitor of SIRT1/2, cambinol, in the progression of *Brucella*. The in vitro results using RAW264.7 cells revealed that cambinol treatment had no effect on adhesion, uptake, intracellular survival and nitric oxide (NO) production during *B. abortus* infection, nor did it directly affect bacterial growth for up to 72 h. Finally, intraperitoneal treatment of 8-week-old female ICR mice infected with *Brucella* showed no differences in the total average weights of spleens and livers; however, the treated mice displayed higher *Brucella* colony-forming units (CFUs) from the spleens. Furthermore, the interleukin (IL)-10 serum level was observed to be lower in treated mice at 7 d post-infection, and none of the cytokines tested showed a change at 14 d post-infection. The overall findings showed that cambinol treatment had no effect on the proliferation of *Brucella* in RAW264.7 macrophages but exacerbated the splenic proliferation of the bacteria in mice and displayed reduced anti-inflammatory cytokine IL-10 at the first week of infection, suggesting that cambinol as an inhibitory of SIRT1/2 could be beneficial in the context of *Brucella* dissemination in animal hosts and that exploration of activating SIRTs could be an alternative treatment against *Brucella* infection.

## 1. Introduction

Brucellosis, caused by several Gram-negative facultative intracellular bacterial species of *Brucella*, is a pervasive zoonotic disease that mainly affects livestock, and it poses significant economic challenges and has considerable public health implications [1,2]. Among the causative agents, *Brucella* (*B.*) *abortus*, *B. melitensis* and *B. suis* are of particular importance in both human and livestock infections across the globe [3]. The disease is one of the most prevalent zoonoses worldwide that is considered to be a priority disease by the World Organization for Animal Health (WOAH), and it is classified as a neglected disease by the World Health Organization (WHO) [4,5]. *Brucella* primarily infects reproductive tissues, lymph nodes and the spleen, with abortion acting as the most clinical manifestation of the disease in animals [5,6]. One of the challenges in the eradication of brucellosis is proper diagnosis; however, several serological tests used for disease diagnosis are often misleading due to possible cross-reactivity [6,7]. Vaccination of livestock remains an essential measure of controlling brucellosis; however, the vaccines have many drawbacks, such as the interference to classical serological diagnosis, risk of infection to humans and virulence recurrence [8,9]. Furthermore, no economically feasible treatment is available for livestock [9]. In humans, the bacteria exploit the host’s immune defenses and establish chronic infections with a range of non-specific clinical manifestations such as fever, fatigue and joint pains, as well as more severe complications, including endocarditis and neurological disorders [1]. No human vaccine is available, and treatment is challenging due to the requirement of prolonged therapy with a combination of antibiotics that are not routinely used for other types of bacterial infections; additionally, resistance of *Brucella* to common antibiotics is annually increasing [10,11].

Sirtuins (SIRTs) are a class of nicotinamide adenine dinucleotide-consuming enzymes which regulate critical signaling pathways, such as in the sustenance of genome integrity, and act as important defense factors against microbial infections in eukaryotic cells [12,13,14]. SIRTs in mammals are involved in cell differentiation and adipogenesis via interactions with peroxisome proliferator-activated receptor (PPAR) in which SIRT1, which is the most well studied among SIRTs, is mostly allocated in the nucleus and functions to repress PPAR, leading to suppression of adipogenesis, lipolysis and fatty acid mobilization, while SIRT2, in the cytoplasm, is involved in regulating metabolism via the protein level’s inhibition in adipocytes to promote adipogenesis [15,16]. When it comes to cancer development, SIRT1 is known to have an inhibitory effect on the p53 tumor suppressor and genes that are involved in the stress response, hence suggesting the role of SIRTs as pro-carcinogenic elements [17]. SIRT1 was also linked to potential therapeutic aspects against lung cancer, as shown by its participation in the induction of apoptosis in lung cells [18]. SIRT2, on the other hand, with its reported ability to modulate cell cycles, could be a target for anticancer drugs due to its level inhibition in human glioma cells being comparable to a tumor-suppressor gene [19]. In microbial infections, SIRT1 is known to be involved in host defense and helps in regulating innate and inflammatory responses, such as in the case of tuberculosis patients and in the pathogenesis of COVID-19, where a low level of the protein correlates with increased levels of proinflammatory cytokines; therefore, this protein is being targeted for interventions in acute and chronic infectious diseases [20]. Furthermore, this protein is reported to participate in orchestrating events for establishing and controlling host defenses during bacterial, viral and parasitic infections [20]. On the other hand, a deficiency of SIRT2 has been reported to enhance bacterial phagocytosis by macrophages as well as survival during chronic staphylococcal infections in a mouse model [17,20,21,22].

Cambinol is a synthetic compound inhibiting the activity of SIRT1 and SIRT2 that is reported to exert antitumor activity [17,23,24]. This synthetic compound was also reported to impair mitogen-activated protein kinase (MAPK) signaling, and this MAPK has been associated with *Brucella* entry upon its full activation [24,25]. Prior to bacterial entry, one critical step is the adhesion to the target cell, and several *Brucella* adhesins have been identified as being involved in the adhesion of *Brucella* to different cell types [26]. To our knowledge, this was the first study to investigate the effect of using cambinol in the context of *B. abortus* infection using macrophages and a mouse model. Nevertheless, we previously reported the potential beneficial effects of SIRT1 activators, such as piceatannol and ginsenoside Rg3, as an alternative approach against *B. abortus* infection via experiments using in vitro and in vivo tests; here, we explored the effects of a synthetic SIRT1 and SIRT2 inhibitor, cambinol, in regard to the ability of *B. abortus* to adhere, invade and grow intracellularly in a murine macrophage cell line while also examining bacterial dissemination and immunoregulation in a mouse model for the purpose of finding alternative treatment against animal brucellosis.

## 2. Materials and Methods

### 2.1. Materials

Cambinol (molecular weight: 360.43 g/mol), 3-(4,5-dimethylthiazol-2-yl)-2,5-diphenyltetrazolium bromide (MTT), 1% penicillin-streptomycin (10,000 U penicillin and 10 mg streptomycin/mL), gentamicin sulfate (50 mg/mL) and dimethyl sulfoxide (DMSO) were purchased from Sigma–Aldrich (Burlington, MO, USA). RPMI 1640 medium, fetal bovine serum (FBS) and gentamicin were purchased from Thermo Scientific (Waltham, MA, USA). Griess reagent was purchased from Promega (Madison, WI, USA) and a BD cytometric bead array (CBA) mouse inflammation kit was purchased from BD Biosciences (Milpitas, CA, USA). Agar was sourced from Yakuri Chemicals Co., Ltd. (Kyoto, Japan) and Brucella broth was obtained from Becton Dickinson (Franklin Lakes, NJ, USA).

RAW264.7 cells (TIB-71, VA, USA) were maintained in fresh medium (RPMI 1640 supplemented with 10% FBS and 1% penicillin-streptomycin) under 5% CO_2_ atmosphere at 37 °C. The cells in the 90–100% confluency were collected using a cell scraper, and an overnight culture with a concentration of 1 × 10^5^ cells per well was used in the experiments. The medium was changed into fresh medium without antibiotics in preparation for all the assays, and the control used consisted of 0.1% DMSO.

*B. abortus* 544 biovar 1 (ATCC 23448) strains were kindly provided by the Laboratory of Bacteriology Division in Animal and Plant Quarantine Agency (Anyang, Republic of Korea) and cultured in Brucella broth with 2% agar at 37 °C for 3 d; one colony was inoculated into 5 mL Brucella broth at 37 °C for 2 d with shaking at 180 rpm. Serial dilutions were performed to determine the number of colony-forming units (CFUs) per mL. Procedures for handling *Brucella* were performed under biosafety facilities and biosafety level 3 practices.

Eight-week-old pathogen-free female ICR mice were purchased from Samtako Bio Co., Ltd. (Osan, Republic of Korea), acclimatized with a provision of ad libitum water and feeds randomly grouped into two of at least six mice per group in metabolic cages (10.5″ W × 19″ L × 8″ H), and were kept at 23 ± 1 °C with a 12 h light/12 h dark cycle. The procedures performed were in compliance with the guidelines and policies established by the Animal Ethical Committee of Jeonbuk National University (NON2022-017-001).

### 2.2. Determination of Cell Viability

An overnight culture of RAW264.7 cells in a 96-well flat-bottom plate was incubated with different concentrations of cambinol (0, 0.15625, 0.3125, 0.625, 1.25, 2.5, 5 and 10 µM) for 48 h. The cells were then washed using PBS and incubated in RPMI 1640 with MTT reagent (5 mg/mL) for 2 h while being protected from light. The medium was carefully removed and incubated in DMSO (150 µL) for 15 min with minimum shaking prior to measuring the absorbance at 540 nm to compute for cell viability.

### 2.3. Determination of Direct Bactericidal Effect

*B. abortus* at a concentration of 2 × 10^4^ colony-forming units (CFUs) per well in a 96-well U-bottom plate was used and incubated at different concentrations of cambinol (0, 0.1, 1 and 10 µM) in PBS for 0, 2, 8, 24, 48 or 72 h at 37 °C. The mixture was centrifuged for 5 s prior to incubation. The mixture was then serially diluted using PBS, plated and incubated at 37 °C for 3 d. Bacterial growth was determined by counting CFUs and expressed in log10.

### 2.4. Adhesion, Internalization and Intracellular Killing Assay

RAW264.7 cells were prepared as that of the cell viability protocol. For adhesion and internalization assays, cells were pre-incubated with different concentrations of cambinol (0, 0.1, 1 and 10 µM) for at least 4 h and then washed with PBS prior to infection at a multiplicity of infection (MOI) of 100. The plate was then centrifuged at 200× *g* for 5 min. For the adhesion assay, the cells were incubated for 0.5 h, washed using PBS and then lysed using distilled water (DW). The lysed cells were then serially diluted using PBS and plated onto the agar. CFUs were determined at 3 d post-incubation of plates at 37 °C. For the internalization assay, cells were incubated for 0 and 0.5 h and then carefully washed with PBS. The cells were then incubated in RPMI 1640 supplemented with 10% FBS and gentamicin (100 µg/mL) for 30 min at 37 °C in a 5% CO_2_ atmosphere. After incubation, the cells were carefully washed with PBS and then lysed using DW. The plating, incubation and counting of CFUs were the same as that of the adhesion assay. For the intracellular killing assay, the cells were pre-infected with *B. abortus* for 1 h before the cells were then carefully washed with PBS prior to incubation with RPMI 1640 supplemented with 10% FBS and gentamicin (100 µg/mL) containing different concentrations of cambinol (0, 0.1, 1 and 10 µM) for 2, 24 and 48 h. After an hour of incubation, the medium was changed with a reduced concentration of gentamicin (30 µg/mL). The washing, plating and CFU determination were the same as that of the adhesion assay.

### 2.5. Nitric Oxide Assay

RAW264.7 cells were prepared, pre-incubated with different concentrations of cambinol (0, 0.1, 1 and 10 µM) and washed with PBS in the same manner as the adhesion assay. Infection with *B. abortus* and prior incubation with different concentrations of cambinol as that of the intracellular killing assay were performed, and the culture medium was collected at 2, 24 and 48 h post-incubation for quantifying nitrite accumulation. Nitric oxide (NO) production was indirectly determined using the Griess Reagent System (Promega, Madison, WI, USA) according to the manufacturer’s instructions.

### 2.6. B. Abortus Infection In Vivo

ICR mice were infected intraperitoneally (IP) with *B. abortus* at 2 × 10^4^ CFU in 100 µL PBS. The mice were then given a once-a-day treatment via an IP route of cambinol (10 µM) or 0.1% DMSO (vehicle) in 100 µL PBS for 5 d and rested for 2 d. IP treatment with cambinol or vehicle was continued once a day for 5 more days before blood was then collected via the tail vein at 7 and 14 d post-infection. The IP treatment of cambinol or vehicle was similar to that performed by Portman et al. [27], although the concentration used in the present study was lower. The animals were observed for any abnormalities in regard to clinical manifestation of disease during the entire treatment period. At 15 d post-infection, mice were then sacrificed via cervical dislocation, and the organs, including the spleen and liver, were then aseptically collected and individually weighed before a 0.05 g part was collected for bacterial proliferation determination. Organs were homogenized in PBS and then serially diluted and plated onto agar. After incubation for 3 d, a bacterial CFU was determined for each gram of the organs.

### 2.7. Cytokine Analysis

The serum was collected and a 50 µL sample was processed for quantification of the different levels of cytokines that are involved during *B. abortus* infection, including L-12p70, TNF-α, IFN-γ, MCP-1, IL-10 and IL-6, using the CBA mouse inflammation kit in accordance with the manufacturer’s instructions. The acquisition of data was performed using a FACSCalibur flow cytometer (BD Biosciences, CA, USA) and then sent to the BD company for measuring cytokine levels.

### 2.8. Statistical Analysis

All the in vitro assays were performed using at least three replicates from three different independent experiments. Data were presented as the means ± SD of all the treatments, and an analysis of the data was conducted using GraphPad InStat software version 3 using a Student’s *t*-test; a value of *p* < 0.05 was considered as statistically significant. Graphs were acquired using GraphPad Prism 5.03 (GraphPad Software, Inc., Solana Beach, CA, USA).

## 3. Results

### 3.1. Effects of Cambinol Treatment in RAW264.7 Cell Viability and B. abortus Growth

**Figure 1:**
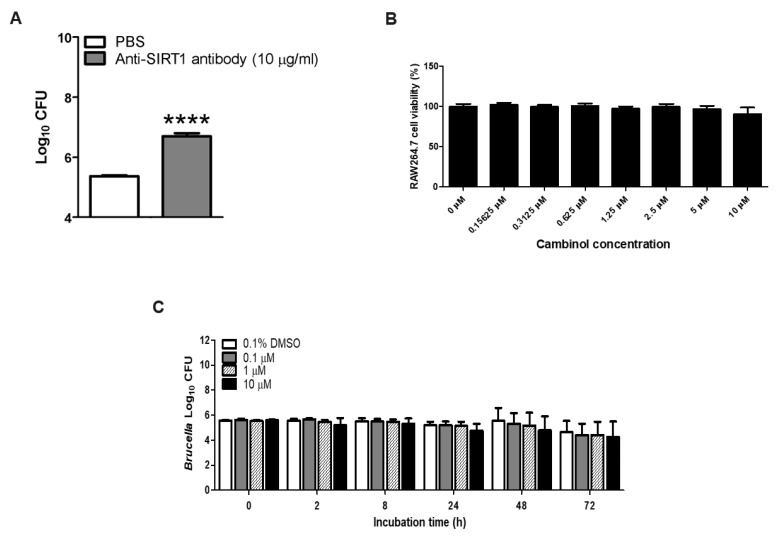
Effect of sirtuin inhibitors in macrophages and *B. abortus*. (**A**) The uptake of *B. abortus* in murine bone marrow-derived macrophages neutralized using mouse anti-sirtuin 1 antibody at 0 h post-infection; (**B**) RAW264.7 cell viability treated with various concentrations of cambinol for 48 h; (**C**) *B. abortus* growth treated with three different concentrations of cambinol at 0, 2, 8, 24, 48 and 72 h. Data are expressed as the mean ± standard deviation, with (****) *p* < 0.0001.

We previously reported the potential beneficial effects of sirtuin activators, including piceatannol and ginsenoside Rg3 [28], and then performed a preliminary investigation of neutralizing sirtuin-1 using a mouse anti-sirtuin-1 antibody in bone marrow-derived macrophages in ICR mice. The result was promising due to the uptake of *B. abortus* in the cells upon pre-incubation with anti-sirtuin-1 antibody being inhibited at 0 h post-infection (Figure 1A). Here, we first determined the highest non-cytotoxic concentration of cambinol. All the concentrations of cambinol used in the present study did not affect the viability of RAW264.7 cells at 48 h post-incubation; hence, 10 µM was the highest concentration used in the entire study (Figure 1B). We tested three different concentrations of cambinol, including the highest non-cytotoxic concentration, and showed that the growth of *B. abortus* was not affected at all incubation time points (Figure 1C).

### 3.2. Effects of Cambinol Treatment During B. abortus Infection in RAW264.7 Cells

**Figure 2:**
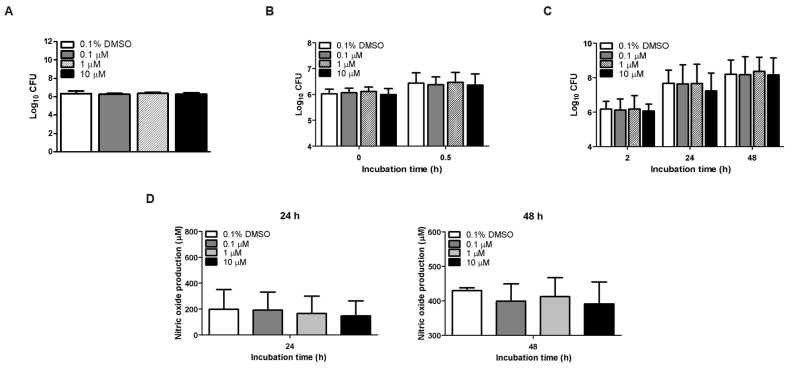
Effect of cambinol treatment in macrophages infected with *B. abortus*. (**A**) Adhesion assay at 0.5 h post-infection; (**B**) internalization assay at 0 and 0.5 h post-infection; (**C**) intracellular killing assay at 2, 24 and 48 h post-incubation; (**D**) nitric oxide assay at 24 and 48 h post-incubation. Data are expressed as the mean ± standard deviation.

*B. abortus* primarily invades its host by adhesion, which is a critical step in this process of infection. In the present study, adhesion of *B. abortus* into RAW264.7 cells was determined upon cambinol treatment at different concentrations, and all the concentrations tested did not affect the ability of the bacteria to adhere to these cells (Figure 2A). Next to adhesion is penetration into host surfaces, and the treatment of cells with cambinol at different non-cytotoxic concentrations did not also affect the uptake of *B. abortus* at 0 and 0.5 h post-infection (Figure 2B). These results suggest that cambinol had no direct effect on the adhesion and internalization of *B. abortus* in regard to RAW264.7 cells (and possibly other phagocytic cells). We then determined the effect of the treatment once the *B. abortus* were inside the host cells, and the results showed that, upon post-incubation at 2, 24 and 48 h, cambinol treatment did not affect the survival of *B. abortus* inside the cells (Figure 2C). We also explored the possible effect of cambinol treatment in the production of nitrite in the cells. The nitrite accumulation was not detectable at 2 h post-incubation during *B. abortus* infection, but the data showed that nitrite accumulation was not statistically different at all concentrations tested at 24 and 48 h post-incubation (Figure 2D).

### 3.3. Effects of Cambinol Treatment During B. abortus Infection in Mice

**Figure 3:**
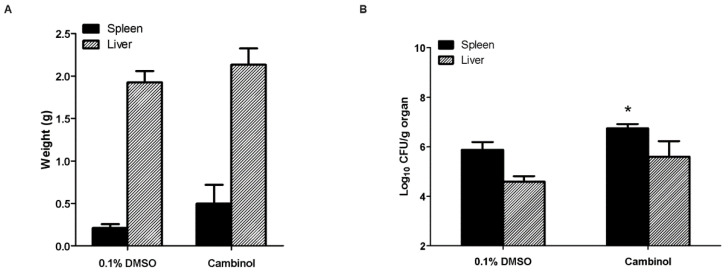
Effect of cambinol after intraperitoneal treatment in ICR mice infected with *B. abortus*. (**A**) Total average organs collected at 15 d post-infection; (**B**) bacterial proliferation in the organs. Data are expressed as the mean (n = 5–6) ± standard deviation, with (*) *p* < 0.05.

No signs of toxicity were observed in mice until the day of sacrifice. The mice that received cambinol treatment displayed slightly higher total average spleen (0.410 ± 0.197 g vs. 0.313 ± 0.136 g) and liver (2.010 ± 0.24 g vs. 1.985 ± 0.159 g) weights as compared with the control group; however, the values were not significantly different (Figure 3A). On the other hand, the cambinol-treated mice exhibited a not quite significant difference in the number of bacterial CFUs expressed in the log10 per gram of the liver (*p* = 0.0575; 5.60 ± 0.601 vs. 4.596 ± 0.213) but, interestingly, did exhibit a significantly higher amount of bacterial proliferation in the spleens (6.751 ± 0.168 vs. 5.870 ± 0.3147) as compared with the control group (Figure 3B).

### 3.4. Effects of Cambinol Treatment in the Serum Cytokine Level in Mice

**Figure 4:**
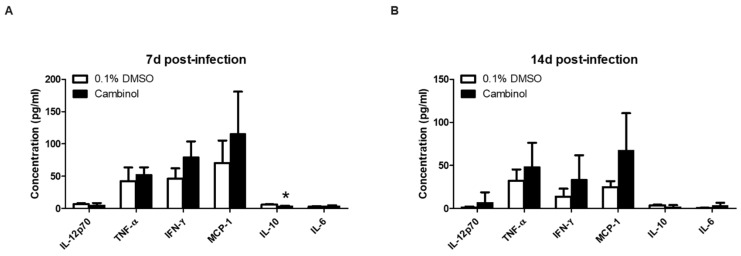
Effect of cambinol treatment in the serum cytokine levels in ICR mice infected with *B. abortus*. (**A**) The different serum cytokine level at 7 d post-infection; (**B**) serum cytokine level at 14 d post-infection. Data are expressed as the mean (n = 5–6) ± standard deviation, with (*) *p* < 0.05.

The mice treated with cambinol at 7 d post-infection displayed a lower serum level of IL-10 (3.660 ± 0.803 pg/mL vs. 5.930 ± 0.797 pg/mL) (Figure 4A). Other cytokines that showed an increase were TNF-α, IFN-γ and MCP-1, but the values were not significant. At 14 d post-infection, none of the cytokines were observed to significantly change (Figure 4B). The IFN-γ/IL-10 ratio was shown to be 1.679 and 18.139 at 7 and 14 d post-infection, respectively, which is suggestive of favoring Th1 immune responses. However, this immune response was accompanied by an increased proliferation of *B. abortus* in the spleens of the mice.

## 4. Discussion

Brucellosis is an important disease as it causes reproductive-related poor performances, resulting in substantial economic losses, and it remains a worldwide zoonotic infectious disease [29,30]. The causative agent is a facultative intracellular bacterium with a predilection regarding different organs of the reticuloendothelial system, including the spleen, liver, lungs, lymph nodes, bone marrow and reproductive tract, and develops strategies to evade innate and adaptive immune responses to promote survival and replication in addition to its intracellular lifestyle [31,32]. *Brucella* mostly reside within professional phagocytic cells, such as macrophages, dendritic cells and neutrophils, and they establish a replicative niche inside the endoplasmic reticulum for subsequent dissemination to other organs [32].

SIRT1 has been reported to contribute to the tuberculosis pathogenesis via attenuating intracellular replication, inducing a phagolysosome fusion and inhibiting persistent inflammatory responses caused by *Mycobacterium tuberculosis*, indicating the potential use of SIRT1 activators in designing effective therapies against tuberculosis [33]. Based on our previous study [28], SIRT1 activators such as piceatannol and ginsenoside Rg3 revealed a promising strategy for controlling *B. abortus* infection via reducing bacterial invasion and intracellular survival within macrophages, and our preliminary study showed that neutralizing sirtuin 1 in murine BMDMs reduced invasion of *B. abortus* into these cells. Here, we tested a murine macrophage cell line of RAW264.7 cells in order to examine the ability of *B. abortus* to adhere, invade and intracellularly replicate via treatment of these cells with cambinol, a SIRT1 and SIRT2 inhibitor.

The first step of the invasion process of *B. abortus* to both professional and non-professional phagocytes involves surface molecular factor interactions between the pathogen and the host cell, leading to bacterial adhesion to the cells [24,26,33]. Adhesin-based vaccines may be a useful strategy in terms of preventing *B. abortus* infection, but the present study showed that cambinol did not affect the ability of *B. abortus* to adhere to phagocytic cell line RAW264.7 cells. A central aspect of the pathogenicity of *Brucella* is the pathogen’s ability to invade, persist and subsequently survive and replicate within several cell types, potentially leading to chronic infections and chronic inflammatory phenomena in different types of tissues [34,35]. The use of sirtuin activators may be beneficial in the control of *B. abortus* infection in phagocytes;, however the use of a SIRT1/2 inhibitor cambinol did not contribute to a more profound *B. abortus* infection, such as in the present study when it comes to internalization capacity and intracellular survival within RAW264.7 cells, as well as with the direct effect on *B. abortus* growth. On the other hand, nitric oxide (NO) induced by IFN-γ could limit inflammation via suppressing *Brucella*-induced inflammasome activation and, in turn, pathology [36]; however, cambinol treatment did not affect the production of nitrite in the cells, which is different to that of the results obtained in a study conducted by Lugrin et al. [25] where macrophages, dendritic cells, splenocytes and whole blood were stimulated with microbes, including heat-inactivated *Escherichia coli* and *Staphylococcus aureus*, and inflammasome. In a study performed by Giordano et al. [17], similar preparation of cambinol was carried out using DMSO, but a concentration of 50 µM was used to treat different cell lines, including MCF-7, NB4 and 3T3-L1, revealing evidence regarding the ability of cambinol to induce differentiations in these cell lines and suggesting its potential contribution to developing an alternative epigenetic therapy that modulates SIRTs for the purpose of counteracting various human pathologies such as tumors and metabolic alterations. Cambinol might have a different mode of action in professional phagocytes, such as RAW264.7 cells, and we suggest an exploration of the potential effect of cambinol treatment in the context of tumor suppression. In fact, in a study conducted by Portmann et al. [27], small molecule SIRT1 inhibitor—cambinol treatment of human hepatocellular carcinoma (HCC) cell lines showed that this agent had a cytostatic effect, including the alteration of cell morphology and cellular senescence. They also found that daily IP injection of cambinol with 100 µL volume of 100 mg/kg five times a week for two weeks did not impair the regenerative capacity of a normal liver but suppressed tumor growth. However, in the present study, cambinol IP treatment displayed a higher *B. abortus* burden or favored bacterial proliferation in the known most-affected organ in mice, which is the spleen. Although cambinol was reported to be beneficial to animals when it comes to developing tumors, the resistance of the animals to intracellular pathogens might be compromised.

Cambinol treatment was shown to inhibit the secretion of TNF, IL-6 and IL-12p40 in a dose-dependent manner and interfere with the gene expression of these cytokines [24]. These proinflammatory cytokines, with the addition of IFN-γ, mediate protective immunity against bacterial infections via activating immune cells in phagocytose, eventually leading to the killing of bacteria, of which TNF plays a crucial role in the early control of *B. abortus* control, while IL-6 is involved activating immune cells and plays a protective role in the immune response against *B. abortus* infection, IL-12 is involved in activating macrophages that can kill intracellular *Brucella* and IFN-γ is involved in immune response to *Brucella* and bacterial clearance [37,38,39,40]. MCP-1, on the other hand, has been reported to serve a major role in attracting immune cells and was observed to be induced during *Brucella* infection [41,42]. In the present study, the mice that were treated with cambinol showed a favorable Th1 immune response, and Th1 is known to be protective against the development of brucellosis and is essential in *Brucella* clearance [43]. However, this was accompanied by increased *B. abortus* burden in the spleens of mice and lower serum cytokine levels of IL-10. In a study performed in human patients with acute *B. abortus* infection, the serum level of IL-10 was significantly increased even after treatment with antibiotics, which could represent a therapeutic opportunity that improves long-term clinical outcomes [44]. Early production of IL-10 was suggested to be beneficial to the pathogen [45] while our previous study also provided data on the suppressive role of IL-10 in phagolysosome fusion and inflammation in response to *B. abortus* infection using murine macrophages [46]. The reduced IL-10 serum level at the first week of treatment after *B. abortus* infection could be beneficial to animals; however, no difference in IL-10 levels was observed at two weeks post-infection, suggesting a different role of cambinol treatment in mice that attenuates the resistance to *B. abortus* infection in mice. This anti-inflammatory cytokine is one of the most important cytokines produced during bacterial infection and is beneficial in helping hosts to survive infections, reduce tissue damage and modulate immune response intensity for successful bacterial clearance [47,48]. However, an attenuated level of IL-10 could be beneficial in the context of *Brucella* dissemination in the present study, such as in the case of *Acinetobacter baumannii* infection in the lungs of the mice where IL-10 protected the mice from the infection and contributed to the bacterial clearance [48]. There is also a possibility of an impaired spleen due to exacerbated *Brucella* burden leading to reduced production of IL-10 since the concentration of the cytokine in the serum represents that of the cytokine produced in the spleens [49,50]. The results in the present study were in agreement with a study conducted by Hajra et al. [51] where inhibition or knocking down of SIRT1 or SIRT3 led to a higher *Salmonella* Typhimurium burden in the liver, spleen and mesenteric lymph node that was attributed to increased bacterial dissemination from the macrophages into the bloodstream due to increased level of serum IL-6. The macrophages fueled by increased fatty acid oxidation and attenuated glycolysis could have facilitated enhanced bacterial proliferation, of which the un-utilized intracellular glucose in the host is readily available to support the pathogen’s own glucose metabolism. These elements could also explain the results in the present study; however, further investigations would be needed in order to prove this pathway. Additionally, a study carried out by Cheng et al. [52] where *M. tuberculosis* intracellular growth was restricted in SIRT1-deficient cells with induced autophagy and a phagosome-lysosome fusion might indicate a possible pathway in the in vivo experiment in the present study. The possibility of insignificant results from the in vitro studies in the present study could be due to the controlled environment in the cells, while the in vivo experiments involved biological complexity, encompassing immune responses, metabolisms and organ interactions.

Overall, our findings suggest that SIRT1 is a promising target in regard to designing effective host-directed therapies for the control of *Brucella* and infections caused by other Gram-negative bacteria, including *Salmonella*, *Acinetobacter* and *Helicobacter.* Further molecular investigations regarding inhibiting SIRT1 or SIRT2 elements during *Brucella* infection or attacks by other Gram-negative microorganisms are encouraged with the use of other control groups, as well as explorations of the down signaling pathway for metabolic regulation, cellular processes, and cancer-focused therapeutic research.

## Data Availability

The original contributions presented in the study are included in the article, further inquiries can be directed to the corresponding author.

## References

[1] Qureshi K.A., Parvez A., Fahmy N.A., Hady B.H.A., Kumar S., Ganguly A., Atiya A., Elhassan G.O., Alfadly S.O., Parkkila S. (2023). Brucellosis: Epidemiology, pathogenesis, diagnosis and treatment-a comprehensive review. Ann. Med..

[2] Najim M.A., Almutawif Y.A., Eid H.M.A., Yousuf A.M., Alahmadi H.A., Alharbi M.E., Aljabri Z.O., Makhdoom H.M., Yoniss M.S., El-Rahim I.H.A.A. (2024). Seroprevalence of brucellosis among high-risk individuals in Madinah, Saudi Arabia. Vet World.

[3] Franc K.A., Krecek R.C., Häsler B.N., Arenas-Gamboa A.M. (2018). Brucellosis remains a neglected disease in the developing world: A call for interdisciplinary action. BMC Public Health.

[4] Khoshnood S., Pakzad R., Koupaei M., Shirani M., Araghi A., Irani G.M., Moradi M., Pakzad I., Sadeghifard N., Heidary M. (2022). Prevalence, diagnosis, and manifestations of brucellosis: A systematic review and meta-analysis. Front. Vet. Sci..

[5] Pinn-Woodcock T., Frye E., Guarino C., Franklin-Guild R., Newman A.P., Bennett J., Goodrich E.L. (2023). A one-health review on brucellosis in the United States. J. Am. Vet. Med. Assoc..

[6] Khan M.Z., Zahoor M. (2018). An Overview of brucellosis in cattle and humans, and its serological and molecular diagnosis in control strategies. Trop. Med. Infect. Dis..

[7] Bonfini B., Chiarenza G., Paci V., Sacchini F., Salini R., Vesco G., Villari S., Zilli K., Tittarelli M. (2018). Cross-reactivity in serological tests for brucellosis: A comparison of immune response of Escherichia coli O157:H7 and *Yersinia enterocolitica* O:9 vs *Brucella* spp. Vet. Ital..

[8] Shi B.C., Li X.Y., Li B., Zheng N.X., Li M., Liu Y., Li C.H., Yan F., He W., Zhao L.Y. (2022). Construction and evaluation of the *Brucella* double gene knock-out vaccine strain MB6 Δbp26ΔwboA (RM6). Zoonoses.

[9] Vives-Soto M., Puerta-Garcia A., Rodriguez-Sanchez E., Pereira J.L., Solera J. (2024). What risk do *Brucella* vaccines pose to humans? A systematic review of the scientific literature on occupational exposure. PLoS Negl. Trop. Dis..

[10] Di Bonaventura G., Angeletti S., Ianni A., Petitti T., Gherardi G. (2021). Microbiological laboratory diagnosis of human brucellosis: An overview. Pathogens.

[11] Ma H.R., Xu H.J., Wang X., Bu Z.Y., Yao T., Zheng Z.R., Sun Y., Ji X., Liu J. (2023). Molecular characterization and antimicrobial susceptibility of human *Brucella* in Northeast China. Front. Microbiol..

[12] Singh C.K., Chhabra G., Ndiaye M.A., Garcia-Peterson L.M., Mack N.J., Ahmad N. (2018). The role of sirtuins in antioxidant and redox signaling. Antioxid. Redox Signal..

[13] Lee S.H., Lee J.H., Lee H.Y., Min K.J. (2019). Sirtuin signaling in cellular senescence and aging. BMB Rep..

[14] Wu Q.J., Zhang T.N., Chen H.H., Yu X.F., Lv J.L., Liu Y.Y., Liu Y.S., Zheng G., Zhao J.Q., Wei Y.F. (2022). The sirtuin family in health and disease. Signal Transduct. Target. Ther..

[15] Picard F., Kurtev M., Chung N., Topark-Ngarm A., Senawong T., De Oliveira R.M., Leid M., McBurney M.W., Guarente L. (2004). Sirt1 promotes fat mobilization in white adipocytes by repressing PPAR-γ. Nature.

[16] Jing E., Gesta S., Kahn C.R. (2007). SIRT2 regulates adipocyte differentiation through FoxO1 acetylation/deacetylation. Cell Metab..

[17] Giordano D., Scafuri B., De Masi L., Capasso L., Maresca V., Altucci L., Nebbioso A., Facchiano A., Bontempo P. (2023). Sirtuin inhibitor cambinol induces cell differentiation and differently interferes with SIRT1 and 2 at the substrate binding site. Biomedicines.

[18] Sun Y., Sun D., Li F., Tian L., Li C., Li L., Lin R., Wang S. (2007). Downregulation of Sirt1 by antisense oligonucleotides induces apoptosis and enhances radiation sensitization in A549 lung cancer cells. Lung Cancer.

[19] Hiratsuka M., Inoue T., Toda T., Kimura N., Shirayoshi Y., Kamitani H., Watanabe T., Ohama E., Candice G.T., Kurimasa A. (2003). Proteomics-based identification of differentially expressed genes in human gliomas: Down-regulation of SIRT2 gene. Biochem. Biophys. Res. Commun..

[20] Kim J.K., Silwal P., Jo E.K. (2022). Sirtuin 1 in host defense during infection. Cells.

[21] Heltweg B., Gatbonton T., Schuler A.D., Posakony J., Li H., Goehle S., Kollipara R., Depinho R.A., Gu Y., Simon J.A. (2006). Antitumor activity of a small-molecule inhibitor of human silent information regulator 2 enzymes. Cancer Res..

[22] Ciarlo E., Heinonen T., Théroude C., Herderschee J., Mombelli M., Lugrin J., Pfefferlé M., Tyrrell B., Lensch S., Acha-Orbea H. (2017). Sirtuin 2 deficiency increases bacterial phagocytosis by macrophages and protects from chronic staphylococcal infection. Front. Immunol..

[23] Manjula R., Anuja K., Alcain F.J. (2021). SIRT1 and SIRT2 activity control in neurodegenerative diseases. Front. Pharmacol..

[24] Bialer M.G., Sycz G., González F.M., Ferrero M.C., Baldi P.C., Zorreguieta A. (2020). Adhesins of *Brucella*: Their roles in the interaction with the host. Pathogens.

[25] Lugrin J., Ciarlo E., Santos A., Grandmaison G., dos Santos I., Roy D.L., Roger T. (2013). The sirtuin inhibitor cambinol impairs MAPK signaling, inhibits inflammatory and innate immune responses and protects from septic shock. Biochim. Biophys. Acta.

[26] de Figueiredo P., Ficht T.A., Rice-Ficht A., Rossetti C.A., Adams L.G. (2015). Pathogenesis and immunobiology of brucellosis: Review of *Brucella*-host interactions. Am. J. Pathol..

[27] Portmann S., Fahrner R., Lechleiter A., Keogh A., Overney S., Laemmle A., Mikami K., Montani M., Tschan M.P., Candinas D. (2013). Antitumor effect of SIRT1 inhibition in human HCC tumor models in vitro and in vivo. Mol. Cancer Ther..

[28] Reyes A.W.B., Kim H., Huy T.X.N., Nguyen T.T., Min W., Lee H.J., Hur J., Lee J.H., Kim S. (2023). Protective effects against *Brucella abortus* 544 infection in a murine macrophage cell line and in a mouse model via treatment with Sirtuin 1 activators resveratrol, piceatannol and ginsenoside Rg3. J. Microbiol. Biotechnol..

[29] Dadar M., Tiwari R., Sharun K., Dhama K. (2021). Importance of brucellosis control programs of livestock on the improvement of one health. Vet. Q..

[30] Tulu D. (2022). Bovine brucellosis: Epidemiology, public health implications, and status of brucellosis in Ethiopia. Vet. Med..

[31] Giambartolomei G.H., Delpino M.V. (2019). Immunopathogenesis of hepatic brucellosis. Front. Cell Infect. Microbiol..

[32] González-Espinoza G., Arce-Gorvel V., Mémet S., Gorvel J.P. (2021). *Brucella*: Reservoirs and niches in animals and humans. Pathogens.

[33] Lopez P., Guaimas F., Czibener C., Ugalde J.E. (2020). A genomic island in *Brucella* involved in the adhesion to host cells: Identification of a new adhesin and a translocation factor. Cell Microbiol..

[34] Baldi P.C., Giambartolomei G.H. (2013). Pathogenesis and pathobiology of zoonotic brucellosis in humans. Rev. Sci. Tech..

[35] Guo X., Zeng H., Li M., Xiao Y., Gu G., Song Z., Shuai X., Guo J., Huang Q., Zhou B. (2023). The mechanism of chronic intracellular infection with *Brucella* spp. Front. Cell Infect. Microbiol..

[36] Lacey C.A., Chambers C.A., Mitchell W.J., Skyberg J.A. (2019). IFN-γ-dependent nitric oxide suppresses *Brucella*-induced arthritis by inhibition of inflammasome activation. J. Leukoc. Biol..

[37] Ritchie J.A., Rupper A., Cardelli J.A., Bellaire B.H. (2012). Host interferon-γ inducible protein contributes to *Brucella* survival. Front. Cell Infect. Microbiol..

[38] Joshi L., Ponnana M., Sivangala R., Chelluri L.K., Nallari P., Penmetsa S., Valluri V., Gaddam S. (2015). Evaluation of TNF-α, IL-10 and IL-6 cytokine production and their correlation with genotype variants amongst tuberculosis patients and their household contacts. PLoS ONE.

[39] Kazemi S., Vaisi-Raygani A., Keramat F., Saidijam M., Soltanian A.R., Alahgholi-Hajibehzad M., Hashemi S.H., Alikhani M.Y. (2019). Evaluation of the relationship between IL-12, IL-13 and TNF-α gene polymorphisms with the susceptibility to brucellosis: A case control study. BMC Infect. Dis..

[40] Guimarães E.S., Martins J.M., Gomes M.T.R., Cerqueira D.M., Oliveira S.C. (2020). Lack of interleukin-6 affects IFN-γ and TNF-α production and early in vivo control of *Brucella abortus* infection. Pathogens.

[41] Tong X., Zeng H., Gu P., Wang K., Zhang H., Lin X. (2020). Monocyte chemoattractant protein-1 promotes the proliferation, migration and differentiation potential of fibroblast-like synoviocytes via the PI3K/P38 cellular signaling pathway. Mol. Med. Rep..

[42] Singh S., Anshita D., Ravichandiran V. (2021). MCP-1: Function, regulation, and involvement in disease. Int. Immunopharmacol..

[43] Yu H., Gu X., Wang D., Wang Z. (2024). *Brucella* infection and Toll-like receptors. Front. Cell Infect. Microbiol..

[44] Tang Y., Ma C., Sun H., Yang S., Yu F., Li X., Wang L. (2021). Serum levels of seven general cytokines in acute brucellosis before and after treatment. Infect. Drug Resist..

[45] Xavier M.N., Winter M.G., Spees A.M., Nguyen K., Atluri V.L., Silva T.M., Bäumler A.J., Müller W., Santos R.L., Tsolis R.M. (2013). CD4^+^ T cell-derived IL-10 promotes *Brucella abortus* persistence via modulation of macrophage function. PLoS Pathog..

[46] Hop H.T., Reyes A.W.B., Huy T.X.N., Arayan L.T., Min W., Lee H.J., Rhee M.H., Chang H.H., Kim S. (2018). Interleukin 10 suppresses lysosome-mediated killing of *Brucella abortus* in cultured macrophages. J. Biol. Chem..

[47] Couper K.N., Blount D.G., Riley E.M. (2008). IL-10: The master regulator of immunity to infection. J. Immunol..

[48] Peñaloza H.F., Noguera L.P., Riedel C.A., Bueno S.M. (2018). Expanding the current knowledge about the role of interleukin-10 to major concerning bacteria. Front. Microbiol..

[49] Kang M.J., Jang A.R., Park J.Y., Ahn J.H., Lee T.S., Kim D.Y., Lee M.S., Hwang S., Jeong Y.J., Park J.H. (2020). IL-10 protects mice from the lung infection of *Acinetobacter baumannii* and contributes to bacterial clearance by regulating STAT3-mediated MARCO expression in macrophages. Front. Immunol..

[50] Athanassakis I., Iconomidou B. (1996). Cytokine production in the serum and spleen of mice from day 6 to 14 of gestation: Cytokines/placenta/spleen/serum. Dev. Immunol..

[51] Hajra D., Rajmani R.S., Chaudhary A.D., Gupta S.K., Chakravortty D. (2024). *Salmonella*-induced SIRT1 and SIRT3 are crucial for maintaining the metabolic switch in bacteria and host for successful pathogenesis. eLife.

[52] Cheng C.Y., Gutierrez N.M., Marzuki M.B., Lu X., Foreman T.W., Paleja B., Lee B., Balachander A., Chen J., Tsenova L. (2017). Host sirtuin 1 regulates mycobacterial immunopathogenesis and represents a therapeutic target against tuberculosis. Sci. Immunol..

